# Breastfeeding, infant formula supplementation, and Autistic Disorder: the results of a parent survey

**DOI:** 10.1186/1746-4358-1-16

**Published:** 2006-09-15

**Authors:** Stephen T Schultz, Hillary S Klonoff-Cohen, Deborah L Wingard, Natacha A Akshoomoff, Caroline A Macera, Ming Ji, Christopher Bacher

**Affiliations:** 1 Division of Epidemiology, Department of Family and Preventive Medicine, School of Medicine, University of California, San Diego, USA; 2 Graduate School of Public Health, San Diego State University, USA; 3 Department of Psychiatry, University of California, San Diego, USA; 4 Autism Internet Research Survey, New Jersey, USA; 5 Dental Corps, United States Navy, San Diego, USA

## Abstract

**Background:**

Although Autistic Disorder is associated with several congenital conditions, the cause for most cases is unknown. The present study was undertaken to determine whether breastfeeding or the use of infant formula supplemented with docosahexaenoic acid and arachidonic acid is associated with Autistic Disorder. The hypothesis is that breastfeeding and use of infant formula supplemented with docosahexaenoic acid/arachidonic acid are protective for Autistic Disorder.

**Methods:**

This is a case-control study using data from the Autism Internet Research Survey, an online parental survey conducted from February to April 2005 with results for 861 children with Autistic Disorder and 123 control children. The analyses were performed using logistic regression.

**Results:**

Absence of breastfeeding when compared to breastfeeding for more than six months was significantly associated with an increase in the odds of having autistic disorder when all cases were considered (OR 2.48, 95% CI 1.42, 4.35) and after limiting cases to children with regression in development (OR 1.95, 95% CI 1.01, 3.78). Use of infant formula without docosahexaenoic acid and arachidonic acid supplementation versus exclusive breastfeeding was associated with a significant increase in the odds of autistic disorder when all cases were considered (OR 4.41, 95% CI 1.24, 15.7) and after limiting cases to children with regression in development (OR 12.96, 95% CI 1.27, 132).

**Conclusion:**

The results of this preliminary study indicate that children who were not breastfed or were fed infant formula without docosahexaenoic acid/arachidonic acid supplementation were significantly more likely to have autistic disorder.

## Background

Autistic disorder (AD), also called autism, is a severe developmental disorder defined by deficits in reciprocal social interaction and communication, and the presence of repetitive and ritualistic behaviors that emerge before three years of age [1]. Some parents report regression in their children or a loss of previously acquired skills with the subsequent development of AD [2]. Parental report of regression in children with AD is estimated to occur in approximately 22% of cases [3]. Recently, parental report of regression has been validated with the use of videotape of children's first and second birthdays [4]. In most cases the cause of AD is unknown [5].

A report by the California Department of Developmental Services shows a noted increase in individuals with a diagnosis of AD receiving services [6]. The proportion (and number) of eligible individuals with AD in their client population of special needs children rose from 3.5% (2,778/80,389) to 12.4% (20,377/163,792) between 1987 and 2002. Changes in case definitions, administrative and diagnostic procedures, and service-related issues have had an effect on the number of eligible individuals with AD in California [7]. The increase in eligible individuals with AD in California may also be due to widening of the case definition to include children with normal or above-normal intelligence [8] or due to diagnostic substitution of children with mental retardation [9].

A world-wide review by Fombonne of autism epidemiological surveys concluded that changes in case definition and improved awareness account for much of the recent increases in autism [5]. A more recent study reported a stable incidence in Midlands, UK over 15 years when study design features were held constant [10]. It is not known whether AD incidence is increasing or whether increases in prevalence are the result of changing diagnostic criteria and better case ascertainment.

The prevalence of breastfeeding in the US increased during the 1970s, decreased during the 1980s, and rose again during the 1990s [11]. For 2002, breastfeeding in the hospital and at six months of age reached an all-time high of 70.1% and 33.2% respectively [11]. Breastfeeding is the recommended method for infant feeding, and increasing the number of mothers who breastfeed their children to six months of age is a goal of Healthy People 2010 in the US [12]. Breastfeeding has been associated with increases in cognitive ability and academic performance [13,14].

Breastfeeding may also be important for the cognitive ability of children at risk for AD. In a study of 145 autistic and 224 normal children, a significantly higher proportion of autistic children (24.8%) compared to control children (7.5%) were weaned by the end of the first week of life [15].

A related study examined the broader category of pervasive developmental disorder and breastfeeding. This study found no significant difference in breastfeeding rates between 50 children with pervasive developmental disorder and 50 control children, although both groups reported significantly less breastfeeding than the national average [16]. Further, the normal siblings of the children with pervasive developmental disorder had breastfeeding rates almost identical to the national average [16]. This study may have been overmatched since cases and controls were matched on IQ which has been linked to breastfeeding and AD [13,14,5].

In 1994, the United Nations and the World Health Organization published a report recommending that infants should be fed breast milk if at all possible, but if fed formula, it should be supplemented with the polyunsaturated fatty acids, docosahexaenoic acid (DHA) and arachidonic acid (ARA) [17]. In January 2002, the first infant formulas supplemented with DHA and ARA were offered for sale in the US, although the older versions of formula without supplementation also continue to be sold [18].

DHA/ARA supplemented formula enhances weight gain in premature infants [19] and raises the plasma and red blood cell concentrations of DHA and ARA in full-term infants to levels comparable to breastfed infants [20]. DHA and ARA are considered conditionally essential substrates during early life and are related to the quality of growth and development [21]. A search of the literature revealed no published studies that have investigated infant formula use in relation to AD.

One study found decreased DHA in the composition of plasma total phospholipids which resulted in significantly lower levels of total omega-3 polyunsaturated fatty acids for autistic compared to mentally retarded subjects [22]. Another study found a significant decrease in the ARA composition of red blood cell polar lipids for children with regressive autism compared to control children [23]. These decreases could be due to decreased availability of DHA and ARA in the diets of these children.

The present study was undertaken for the purpose of determining whether breastfeeding or the use of infant formula (with or without DHA/ARA supplementation) is associated with AD. The hypothesis is that less breastfeeding and use of infant formula without DHA/ARA supplementation increase the likelihood of AD.

## Methods

The Autism Internet Research Survey was created by the parent of a child with autism hoping to identify possible causes for the rise in autism. The survey did not state whether the rise in autism was due to a rise in incidence or in the number of individuals registered for special education programs; however, parents who believe there is an increase in autism incidence may have been more inclined to take the survey. In order to quickly obtain the number of cases required for this analysis, the internet was used to solicit participants. Subsequently, this developed into a New Jersey-based nonprofit organization, Autism Internet Research Survey. Neither the organization nor the survey is related to any commercial entity.

The Autism Internet Research Survey invited parents to complete surveys for their children with or without AD. Whether a child had AD was self-reported by clicking on one of two links: "For those with autistic children who want to take the survey click here." or "For those who want to take the control survey (you have children, but not with any autism spectrum disorder) click here."

Ads for the surveys were placed online using Google and restricted to the United States. Individuals who performed online searches containing keywords (autistic, autism research, autism, MMR, autism education, etc.) were shown an ad requesting their participation in a research survey. The total number of keywords used was 306, and they were grouped into the following categories: autism and autistic features 262, treatment for autism 24, prominent people involved in autism 13, and possible causes of autism 7. Participants completed the surveys from February to April 2005. The surveys included 91 questions on breastfeeding, infant formula use, date of birth, and the nature of their child's development. Limiting the age range to children two to 18 years and the respondents to parents yielded 861 case and 123 control children.

Breastfeeding data was recorded from a drop-down menu with nine choices of duration of breastfeeding. This variable was recoded into five categories: none, less than 2 months, 2–6 months, more than 6 months, and unknown. These breastfeeding categories were tested for association with autism using logistic regression.

Infant formula use data was recorded from a drop-down menu with 39 brand-name choices as well as "Other", "None", and "I don't know". This variable was recoded into three categories: None, Formula without DHA/ARA, and Formula with DHA/ARA. Information regarding DHA/ARA supplementation was ascertained from the manufacturers websites. If parents chose the category "Other" or "I don't know", no determination could be made regarding DHA/ARA supplementation, and the data was excluded from further analysis (n = 38). The remaining three infant formula categories were tested for association with autism using logistic regression.

Children under two years old were excluded since AD is rarely diagnosed before age two. For analysis of breastfeeding, the age range was limited to 2–18 years. Eighteen years was chosen as the upper age for the range in an attempt to minimize recall bias from the parents.

For analysis of infant formula, 2–4 years was chosen as the age range. Four years was chosen as the upper age for this portion of the study since supplementation with DHA/ARA has only been available in the US since 2002. Children older than four would not have had the opportunity to use DHA/ARA supplemented formulas during the first year of life.

Parents of autistic children were also questioned about the nature of their child's development. Three choices were given in a drop-down menu: 1) My child developed normally, then regressed (lost skills). 2) My child developed normally, then stopped. 3) My child never developed in a normal way. For the purposes of this study, if response number 1 was chosen, the child was assumed to have a regression in development, i.e. lost skills that had previously been acquired.

In order to remove the effects of congenital conditions associated with autism from the odds ratios seen in this study, breastfeeding and infant formula use were also tested for association with autism for the subset of children with reported regression in development.

All analyses, including characterization of the population and logistic regression, were performed using SAS version 9.1 for Windows (SAS Institute Inc., Cary, North Carolina). This study was approved by the University of California, San Diego Human Research Protections Program and the Institutional Review Board at San Diego State University.

## Results

Table 1 presents the characteristics of children in the Autism Internet Research Survey. For those aged 2–18 years, there were 861 cases and 123 controls, and for those aged 2–4 years, there were 150 cases and 38 controls. Parental report of regression in development for these two age groups was 25% and 23% respectively.

**Table 1:** Characteristics of participants in the Autism Internet Research Survey 2005.

	Cases	Controls
For children aged 2–18 years:	N = 861	N = 123
	Mean (SD)	Mean (SD)
Age (years)	7.8 (3.9)	7.4 (4.5)
	%	%
No breastfeeding	28	16
Breastfeeding <2 months	23	20
Breastfeeding 2–6 months	19	22
Breastfeeding >6 months	25	36
Unknown	5	6
Parental report of regression in their child's development	25	---
For children aged 2–4 years:	N = 150	N = 38
	Mean (SD)	Mean (SD)
Age (years)	3.2 (0.5)	3.0 (0.6)
	%	%
Exclusive breastfeeding (no infant formula use)	8	16
Infant formula with DHA/ARA	26	58
Infant formula without DHA/ARA	43	18
Other infant formula (DHA/ARA content unknown)	17	3
Unknown	6	5
Parental report of regression in their child's development	23	---

For children aged 2–18 years, the mean age of cases and controls was similar at 7.8 and 7.4, respectively. Breastfeeding varied by group, with no breastfeeding being reported more frequently for cases (28%) than controls (16%) and breastfeeding for greater than six months being reported more frequently for controls (36%) than cases (25%).

For children aged 2–4 years, the mean age of cases was 3.2 and of controls was 3.0. Infant formula use varied by group with exclusive breastfeeding (no formula use) being reported more often for controls (16%) than cases (8%). Use of infant formula containing DHA/ARA was also reported more frequently for controls (58%) than cases (26%) while use of infant formula without DHA/ARA was reported more frequently for cases (43%) than controls (18%). Breastfeeding for greater than six months was reported for all children with no formula use and 23% of children with formula use (data not shown).

Age-adjusted associations of breastfeeding and AD are presented in Table 2 for children aged 2–18. Decreased breastfeeding was significantly associated with increased likelihood of having a child with AD. No breastfeeding versus breastfeeding for more than six months was significantly associated with an increase in the odds of having AD when all cases were considered (OR 2.48, 95% CI 1.42, 4.35) and after limiting cases to children with regression in development (OR 1.95, 95% CI 1.01, 3.78). Duration of breastfeeding showed a dose-response relationship with AD before and after limiting cases to children with regression in development (chi square test for trend, p = 0.0007 and p = 0.031 respectively).

**Table 2:** Age-adjusted associations of breastfeeding and autistic disorder for children aged 2–18 years.

Variable	Odds Ratio	(95% Confidence Interval)	p value
No Breastfeeding	2.48	(1.42 – 4.35)	0.001
Breastfeeding <2 months	1.70	(1.00 – 2.88)	0.050
Breastfeeding 2–6 months	1.27	(0.75 – 2.14)	0.373
Breastfeeding >6 months	reference		
Limited to cases with reported regression in development			
No Breastfeeding	1.95	(1.01 – 3.78)	0.048
Breastfeeding <2 months	1.84	(0.98 – 3.44)	0.057
Breastfeeding 2–6 months	1.59	(0.86 – 2.96)	0.141
Breastfeeding >6 months	reference		

Age-adjusted associations of infant formula use with AD are presented in Table 3 for children 2–4 years old. Use of infant formula without DHA/ARA supplementation versus exclusive breastfeeding was associated with a significant increase in the odds of AD when all cases were considered (OR 4.41, 95% CI 1.24, 15.7) and after limiting cases to children with regression in development (OR 12.96, 95% CI 1.27, 132). Use of unsupplemented versus supplemented infant formula was also associated with a significant increase in the odds of AD (OR 4.33, 95% CI 1.65, 11.4) when all children were considered and after excluding children who were breastfed for more than six months (OR 4.78, 95% CI 1.57, 14.6) (data not shown in table). Use of infant formula with DHA/ARA compared to exclusive breastfeeding was not significantly associated with an increase in the odds of AD.

**Table 3:** Age-adjusted association of infant formula use with autistic disorder for children aged 2–4 years.

Variable	Odds Ratio	(95% Confidence Interval)	p value
Formula without DHA/ARA	4.41	(1.24 – 15.7)	0.022
Formula with DHA/ARA	1.02	(0.33 – 3.18)	0.977
Exclusive breastfeeding (no formula use)	reference		
Limited to cases with reported regression in development			
Formula without DHA/ARA	12.96	(1.27 – 132)	0.031
Formula with DHA/ARA	2.76	(0.28 – 27.4)	0.387
Exclusive breastfeeding (no formula use)	reference		

## Discussion

The children with AD in this survey were significantly less likely to have been breastfed and were significantly less likely to have been fed infant formula with DHA/ARA than typically developing children. A possible mechanism for these associations is immune system dysfunction. Without breast milk or infant formula supplemented with DHA/ARA, some children's immune systems could be compromised which could in theory lead to AD. Breast milk provides the infant IgA and other humoral components from the mother which are important for the immune protection of the infant. Also, use of formula with DHA/ARA supplementation could be beneficial to the infant immune system. DHA and ARA are discussed in a review by Yaqoob as important for proper immune system functioning [24].

The results of this study are from an online internet survey and should be viewed with caution. This survey was not a random sampling of the population and has the attendant problem of ascertainment bias. Only individuals who had computers and were interested in taking an online survey were participants. Also, the survey could have biased participant responses by telling them the purpose was to find reasons for the rise in autism.

The present study relied on self-reported data regarding the diagnosis of AD and therefore the accuracy of diagnosis was not confirmed. However, parental report of the proportion of cases with regression in development in this study (23% for children aged 2–4 and 25% for children aged 2–18) was similar to that seen in a study by Siperstein and Volkmar in which 22% of parents reported regression in their children diagnosed with AD using DSM-IV criteria [3]. However, the present study did not use the same question regarding regression, and the similarities in the proportion with reported regression may be due to other reasons.

The exposure data of breastfeeding and infant formula use were of necessity also self-reported. Reliance on self-report leads to misclassification bias; however, there is no reason to believe that this bias is differential, especially in terms of formula use with and without supplementation, and is therefore assumed to be random. Non-differential misclassification would bias the results toward the null, indicating the odds ratios seen in this study could be higher in a more rigorous study.

The internet survey was a parent-based effort and did not include all of the demographic questions normally found in epidemiologic surveys. The analyses in this study were only adjusted for age. Information on gender and socioeconomic status (SES) was not obtained. SES has been shown to not be associated with AD; however, gender is associated with AD – approximately 76% of those with AD are male [25]. Gender could confound the association of breastfeeding and AD if mothers are more likely to breastfeed due to the child's gender, but child's gender has been shown to not be associated with duration of breastfeeding [26]. The health status of the controls is unknown, other than the parents indicating that their children had no autism spectrum disorder. These questions will need to be addressed in future studies.

The survey used for this study also did not address the reason why some mothers stopped or did not initiate breastfeeding. If there is a problem breastfeeding infants due to AD, then this is a source of possible confounding; however, this would not be an issue for the infant formula analysis. Also, the infant formula information used in this study did not categorize the amounts of partial infant formula use. These questions will need to be addressed in a future study.

One advantage of using this internet survey was the speed at which the survey was administered and the results received. This survey was completed in less than three months. Also this survey had a large number of participants, 861 cases and 123 controls, which gives the study sufficient power to detect differences in breastfeeding and infant formula use. This was an innovative study produced by concerned parents who want to find answers to the question of what causes AD.

Another advantage is the large number of infant formula choices in the survey's drop-down menu – 39 brand name choices were available along with "none", "other", and "I don't know". Having this number of infant formula choices allowed this variable to be accurately recoded into categories with and without DHA/ARA supplementation. Interestingly, 17% of case parents chose the "other" category compared to 3% of control parents. This indicates that a larger percent of case parents could not find their infant formula brand on the drop-down menu. The reason for this difference is unknown; however, if all of the children in the "other" category used supplemented formula, then control children still used more supplemented formula than cases, and the association between lack of supplementation and autistic disorder remains significant (OR 4.39, 95% CI 1.23, 15.7). Alternatively, if all of those in the "other" category used formula not supplemented with DHA/ARA, then the association between autistic disorder and lack of supplementation is even greater (OR 5.64, 95% CI 1.64, 19.4).

## Conclusion

While Tanoue and Oda [15] found a significantly higher number of children with autism compared to control children had already stopped breastfeeding when assessed at the end of the first week of life, the present study is the first to show that increased duration of breastfeeding is associated with a decreased likelihood of AD. This is also the first study to suggest a possible link between the use of infant formula without DHA/ARA supplementation and AD. However, this study was based on a small group and should be followed by a larger more rigorous study to confirm the results.

## Competing interests

The author(s) declare that they have no competing interests.

## Authors' contributions

SS participated in the design, performed the analyses, and helped draft the manuscript. CB participated in the design and performed the survey. MJ participated in the design and consulted on statistical analyses. HK-C, DW, CM, and NA participated in the design and helped draft the manuscript.
